# Exploring the Association Between Physical Activity and Risk of Mental Health Disorders in Saudi Arabian Adults: Cross-sectional Study

**DOI:** 10.2196/25438

**Published:** 2021-04-14

**Authors:** Nora A Althumiri, Mada H Basyouni, Nasser F BinDhim

**Affiliations:** 1 Sharik Association For Health Research Riyadh Saudi Arabia; 2 Ministry of Health Riyadh Saudi Arabia; 3 College of Medicine Alfaisal University Riyadh Saudi Arabia; 4 Saudi Food and Drug Authority Riyadh Saudi Arabia

**Keywords:** Saudi Arabia, physical activity, mental health, depression, anxiety, risk, symptoms, cross-sectional, survey

## Abstract

**Background:**

The relationship between physical activity and mental health, especially the symptoms of major depressive disorder (MDD) and generalized anxiety disorder (GAD), has received increasing attention in recent years.

**Objective:**

The aim of this study was to explore the association between fulfilling the World Health Organization (WHO) global recommendations on physical activity and the risk and symptoms of MDD and GAD in the Saudi population.

**Methods:**

This study was a secondary analysis of data from a large nationwide cross-sectional survey conducted via phone interviews in June and July 2020. In this study, a proportional quota sampling technique was used to obtain an equal distribution of participants, stratified by age and gender, across the 13 regions of Saudi Arabia. The main mental health screening tool used for the risk of MDD was the Patient Health Questionnaire-9 (PHQ-9). Risk of GAD was measured using the Generalized Anxiety Disorder-7 (GAD-7) scale. Participants self-reported whether they fulfill the WHO global recommendations on (1) moderate-intensity aerobic physical activity (MIPA) and (2) vigorous-intensity aerobic physical activity (VIPA). The results were then analyzed based on the following two categories: fulfilling the WHO global recommendations or not.

**Results:**

The data analysis included 8333 participants recruited in the main study between June and July 2020. The response rate was 81.45% (8333/10,231). Of them, 50.3% (4192/8333) were female, and the mean age was 36.5 years, with a median age of 36 years and a range from 18 to 90 years. The average total PHQ-9 score was 5.61, and the average total GAD-7 score was 4.18. For men, the average total PHQ-9 and GAD-7 scores were associated with fulfilling recommendations for MIPA; however, there were no associations for VIPA in both sexes. Fulfilling the WHO’s recommendations for MIPA was associated with considerably fewer depressive symptoms in six of the nine items in the PHQ-9. Moreover, fulfilling recommendations for MIPA was associated with considerably fewer anxiety symptoms in six of the seven items in the GAD-7. However, fulfilling recommendations for VIPA was significantly associated with more depressive symptoms in one of the PHQ-9 items (“Thoughts that you would be better off dead or thoughts of hurting yourself in some way;” *P*<.001).

**Conclusions:**

This study has shown that fulfilling guidelines on MIPA is associated with less overall risk of MDD and GAD in males and fewer depressive and anxiety symptoms generally in a nonclinical population. In the general population, an increase in MIPA may improve well-being and general mental health.

## Introduction

The World Health Organization (WHO) has recognized health as “A state of complete physical, mental, and social well-being and not the absence of disease or weakness” [1]. Globally, poor mental health and well-being are considered a major cause of disease, with depression considered a leading contributor [2]. According to the WHO, one in four people globally is affected by a mental disorder at some point in their life, which means 450 million people currently have such conditions [3].

Physical activity is recognized as a key factor in the prevention and management of mental illness, including, but not limited to, mental disorders such as major depressive disorder (MDD), generalized anxiety disorder (GAD), and posttraumatic stress disorder [2]. Increased physical activity can be used as a complementary strategy with other treatment modalities to prevent and manage mental health conditions, as it can delay onset and reduce a wide range of symptoms [4]. However, the WHO’s recommendation for physical activity is that adults should participate in at least 150 minutes of moderate-intensity aerobic physical activity (MIPA) per week, 75 minutes of vigorous-intensity aerobic physical activity (VIPA) per week, or an equivalent combination of the two [5]. According to the WHO, the Eastern Mediterranean Region has the highest prevalence of physical inactivity (35%), and Saudi Arabia has the highest rate of physical inactivity among the Gulf Cooperation Council countries [6]. In particular, a recent study found that the prevalence of physical inactivity in Saudi Arabia (not meeting the WHO recommendations) ranged between 66.8% and 81.2%. Females and males did not differ in the frequency of physical activity [7].

A recent meta-analysis of prospective cohort studies demonstrated in subgroup analyses that depression could be reduced by 22% if participants complete 150 minutes per week of MIPA [8]. Despite this, the study was not able to describe the relationship between physical activity and mental health due to the small sample size; however, it was concluded that higher levels of physical activity were associated with a lower risk of developing depression [8]. A systematic review study found that a light amount of physical activity (<150 min/week) was also associated with a reduced likelihood of depression [9]. It is important to know whether mental health benefits can be obtained with a lower level of physical activity, particularly among those at risk of mental illness, as well as those who prefer light physical activity to MIPA [2]. However, a study conducted between July 2012 and June 2014 at psychiatric clinics in five regions of Saudi Arabia showed that higher rates of physical activity were positively correlated with primary bipolar disorders and use of antianxiety medications but negatively correlated with primary anxiety disorders, use of antidepressant medications, and use of multiple psychotropic medications [10]. However, these two studies were in hospital and university settings. No study has characterized physical activity and mental health in Saudi Arabia in community settings with national-level coverage. More studies are needed to characterize physical inactivity in Saudi Arabia and its associations with the risk of mental health disorders and mental health symptoms, as different symptoms have different risk factors and impairments [11]. Thus, the aim of this study was to explore the association between fulfilling the WHO global recommendations on physical activity and the risks and symptoms of MDD and GAD in the Saudi population.

## Methods

### Design

This study was a secondary analysis of a multiwave, cross-sectional, national-level mental health screening (Saudi Mental Health Surveillance System) completed via computer-assisted phone interviews conducted in two waves between June and July 2020. The full methodology and rationale were previously published as a study protocol article [12].

### Participants and Recruitment

Adults aged 18 years or above from Saudi Arabia were recruited via a random phone number list generated from the Sharik Association for Health Research, a research participant database [13]. The Sharik database includes individuals interested in participating in health research, currently has more than 70,000 potential participants, is growing daily, and covers the 13 administrative regions of Saudi Arabia [13].

### Sample Size

This surveillance system used a proportional quota sampling technique to achieve an equal distribution of participants, stratified by age, gender, and region within and across the 13 administrative regions of Saudi Arabia. The Saudi Mental Health Surveillance System uses two age groups based on Saudi Arabia’s median adult age of 36 years. This led to a quota of 52 for this study.

The sample size was calculated based on the depth of the subanalysis needed for the surveillance system, which compares age and gender groups across regions with a medium effect size of approximately 0.3, 80% power, and a 95% CI [14]. Therefore, each quota group required 78 participants and a total sample of 312 per region for a grand total of 4056 participants per wave. Once the quota sample was reached, participants with similar characteristics were not eligible to participate in the study. The sampling process was controlled automatically by the data collection system with no human interference [15].

### Variables

The data used in this secondary analysis included general demographic variables, such as age, gender, and region, and other health-related variables, such as a history of mental health conditions and physical activity.

Physical activity was assessed by asking the participants on how many days they performed the recommended levels, duration, and intensity of physical activities within the previous week, using two brief assessment tools for physical activity. For VIPA, the question was “how many times a week do you usually do 20 minutes or more of vigorous-intensity physical activity that makes you sweat or puff and pant?” (eg, heavy lifting, digging, jogging, aerobics, and fast bicycling). For MIPA, the question was “how many times a week do you usually do 30 minutes or more of moderate-intensity physical activity or walking that increases your heart rate or makes you breathe harder than normal?” (eg, carrying light loads, bicycling at a regular pace, and doubles tennis) [16]. The answers for these two questions ranged from 0 days in the last week to 7 days in the last week. The main mental health screening tool used for the risk of MDD was the Patient Health Questionnaire (PHQ-9) [17-20]. Risk of GAD was measured using Generalized Anxiety Disorder-7 (GAD-7) [21].

### Outcome Measures

To categorize the participants’ physical activity, this study used the WHO’s global recommendations on physical activity for adults (18-64 years old), which are in line with guidelines from the Centers for Disease Control and Prevention and the American Heart Association [22,23] as follows: (1) VIPA, 75 minutes per week or (2) MIPA, 150 minutes per week. Based on participants’ self-reported responses to the interview questionnaire (ie, number of exercise minutes, frequency, and intensity level per week), two categorical outcome variables were created that reflect whether guidelines were met as follows: MIPA (1, at least 150 minutes of MIPA per week; 0, less than 150 minutes) and VIPA (1, at least 75 minutes of VIPA per week; 0, less than 75 minutes).

The PHQ-9 score is the total score of nine questions, each of which is answered on a 4-point Likert scale ranging from 0 to 3, for a final total score between 0 and 27. Similarly, the GAD-7 score is calculated as the total score of seven questions, each of which is answered on a 4-point Likert scale ranging from 0 to 3, for a final total score between 0 and 21.

### Statistical Analysis

Quantitative variables are presented by mean if they have a normal distribution or by median and range, as appropriate, and compared using the *t* test. Qualitative variables are presented as percentages and CIs and compared using Pearson chi-square test. As this study used automated electronic data collection, there are no missing values. The QPlatform also includes a data integrity check to prevent users from entering invalid data [15].

### Ethical Considerations

Ethical approval was obtained from the Sharik Association for Health Research institutional review board (approval number 01-2020).

## Results

The data set included 8333 participants from two waves (June and July 2020). The response rate was 81.4% (8333/10,231). The mean age was 36.5 years, with a median age of 36 years and a range from 18 to 90 years. Table 1 shows the main participant characteristics.

**Table 1:** Participant characteristics.

Characteristic		Value (N=8333), n (%)
**Gender**		
	Male	4141 (49.7)
	Female	4192 (50.3)
**Region**		
	Asir	643 (7.7)
	Baha	625 (7.5)
	Eastern Region	645 (7.7)
	Hail	646 (7.8)
	Jazan	645 (7.7)
	Al Jouf	638 (7.7)
	Madinah	641 (7.7)
	Makkah	648 (7.8)
	Najran	643 (7.7)
	Northern Boarder	639 (7.7)
	Qassim	648 (7.8)
	Riyadh	643 (7.7)
	Tabuk	629 (7.5)
**Previously diagnosed with major depressive disorder**		
	Yes	191 (2.3)
	No	8142 (97.7)
**Previously diagnosed with generalized anxiety disorder**		
	Yes	130 (1.6)
	No	8203 (98.4)

The average total PHQ-9 score was 5.61, and the average total GAD-7 score was 4.18. As shown in the *t* test analysis presented in Table 2 and Table 3, the average total PHQ-9 and GAD-7 scores were associated with fulfilling the recommendation for MIPA but not for VIPA.

**Table 2:** Independent samples *t* test of the association of moderate-intensity aerobic physical activity with the Patient Health Questionnaire-9 and Generalized Anxiety Disorder-7 total scores.

Variable	150+ min MIPA^a^/week		*t* (df)	*P*
	No (n=6961)	Yes (n=1372)		
Mean PHQ-9^b^ score	5.69	5.15	3.96 (8331)	.001
Mean GAD-7^c^ score	4.33	3.95	3.06 (8331)	.002

^a^MIPA: moderate-intensity aerobic physical activity.

^b^PHQ-9: Patient Health Questionnaire-9.

^c^GAD-7: Generalized Anxiety Disorder-7.

**Table 3:** Independent samples *t* test of the association of vigorous-intensity aerobic physical activity with the Patient Health Questionnaire-9 and Generalized Anxiety Disorder-7 total scores.

Variable	75+ min VIPA^a^/week		*t* (df)	*P*
	No (n=7326)	Yes (n=1007)		
Mean PHQ-9^b^ score	5.63	5.40	1.48 (8331)	.137
Mean GAD-7^c^ score	4.28	4.20	0.50 (8331)	.614

^a^VIPA: vigorous-intensity aerobic physical activity.

^b^PHQ-9: Patient Health Questionnaire-9.

^c^GAD-7: Generalized Anxiety Disorder-7.

In terms of the differences between males and females, in the PHQ-9 score for males, there were significant differences with MIPA (*t*_4139_=3.65, *P*<.001), but not with VIPA (*t*_4139_=1.21, *P*=.23). However, for females, there were no significant differences with MIPA (*t*_4190_=1.31, *P*=.19) or VIPA (*t*_4190_=−0.16, *P*=.87).

In the GAD-7 score for males, there were significant differences with MIPA (*t*_4139_=2.38, *P*=.02), but not with VIPA (*t*_4139_=0.26, *P*=.79). However, for females, there were no significant differences with MIPA (*t*_4190_=1.43, *P*=.15) or VIPA (*t*_4190_=−0.39, *P*=.69).

MIPA was associated with considerably fewer depressive symptoms in six of the nine items in the PHQ-9 (Table 4). Moreover, MIPA was associated with considerably fewer anxiety symptoms in six of the seven items in the GAD-7 (Table 5). However, none of the anxiety symptoms (GAD-7 items) was associated with VIPA. On the other hand, VIPA was associated with fewer depressive symptoms in three of the nine items in the PHQ-9, including “Little interest or pleasure in doing things” (*P*=.02), “Trouble falling or staying asleep, or sleeping too much” (*P*=.008), and “Feeling tired or having little energy” (*P*=.01). However, VIPA was associated with more depressive symptoms in one of the PHQ-9 items (“Thoughts that you would be better off dead or thoughts of hurting yourself in some way;” *P*<.001).

**Table 4:** Independent samples *t* test of the association between moderate-intensity aerobic physical activity and depressive symptoms (Patient Health Questionnaire-9 items).

PHQ-9^a^ items	150+ min MIPA^b^/week^c^		*t* (df)		*P*
	No (n=6961)	Yes (n=1372)			
Little interest or pleasure in doing things	0.79	0.67	4.78 (8331)	<.001	
Feeling down, depressed, or hopeless	0.81	0.70	4.64 (8331)	.001	
Trouble falling or staying asleep, or sleeping too much	0.95	0.93	1.03 (8331)	.30	
Feeling tired or having little energy	0.96	0.88	3.06 (8331)	.002	
Poor appetite or overeating	0.72	0.68	1.57 (8331)	.12	
Feeling bad about yourself — or that you are a failure or have let yourself or your family down	0.46	0.42	1.65 (8331)	.10	
Trouble concentrating on things, such as reading the newspaper or watching television	0.47	0.43	2.02 (8331)	.044	
Moving or speaking so slowly that other people could have noticed? Or the opposite — being so fidgety or restless that you have been moving around a lot more than usual	0.37	0.32	2.82 (8331)	.005	
Thoughts that you would be better off dead or thoughts of hurting yourself in some way	0.17	0.13	2.89 (8331)	.004	

^a^PHQ-9: Patient Health Questionnaire-9.

^b^MIPA: moderate-intensity aerobic physical activity.

^c^The scores of PHQ-9 items are provided.

**Table 5:** Independent samples *t* test of the association between moderate-intensity aerobic physical activity and anxiety symptoms (Generalized Anxiety Disorder-7 items).

GAD-7^a^ items	150+ min MIPA^b^/week^c^			*t* (df)		*P*
	No (n=6961)	Yes (n=1372)				
Feeling nervous, anxious, or on edge	0.77	0.72	2.33 (8331)		.02	
Not being able to stop or control worrying	0.54	0.46	3.56 (8331)		<.001	
Worrying too much about different things	0.61	0.55	2.28 (8331)		.02	
Trouble relaxing	0.70	0.64	2.27 (8331)		.02	
Being so restless that it’s hard to sit still	0.41	0.36	2.75 (8331)		.006	
Becoming easily annoyed or irritable	0.80	0.77	1.10 (8331)		.27	
Feeling afraid, as if something awful might happen	0.50	0.44	2.70 (8331)		.007	

^a^GAD-7: Generalized Anxiety Disorder-7.

^b^MIPA: moderate-intensity aerobic physical activity.

^c^The scores of GAD-7 items are provided.

### Data Availability Statement

The data are available from Sharik Association for Health Research upon request.

## Discussion

### Principal Findings

This study explored the association between WHO global recommendations on physical activity for mental health and the risks of MDD and GAD in a Saudi community population sample. It is one of the first and largest studies to explore the association between physical activity and risk of mental health disorders (MDD and GAD) in general population settings in Saudi Arabia. The study found that the average total PHQ-9 and GAD-7 scores were associated with fulfilling recommendations for MIPA among men only; however, there were no associations for VIPA in both sexes. Further analysis showed that the associations of fulfilling MIPA with PHQ and GAD were only significant for males. MIPA was associated with considerably fewer depressive symptoms in six of the nine items in the PHQ-9. Moreover, MIPA was associated with considerably fewer anxiety symptoms in six of the seven items in the GAD-7 scale. None of the anxiety symptoms (GAD-7 items) was associated with VIPA. On the other hand, VIPA was associated with considerably fewer depressive symptoms in three of the nine items in the PHQ-9. However, VIPA was associated with more depressive symptoms in the self-harm and suicide ideation item of the PHQ-9.

MIPA has been shown to have a significant association with better mental health in several other studies across many countries, confirming its cross-cultural effectiveness [2,4,8]. According to recent meta-analysis study findings, MIPA has significant effects on the severity of depressive symptoms among nonclinical populations (age >18 years) after supervised or unsupervised training. This effect of even low-intensity physical activity may be attributable to the exercise-induced release of neurotrophic growth factors that are responsible for nerve growth and synaptic plasticity in the brain [24]. However, other studies found different effects of physical activity on mental health in relation to gender [26].

VIPA is generally associated with better mental well-being, including coping, autonomy, and personal growth [26,27]. However, some evidence suggests that VIPA may have no association with depression and anxiety, although VIPA is associated with better scores for some depressive symptoms [27-30]. One explanation is that VIPA may be associated with more negative affective states during participation, compared with MIPA, which can predict dropout and therefore reduce the likelihood of meeting physical activity guidelines [2,31]. In addition, the strenuous nature of VIPA may also undermine competence, particularly for those who are inactive [2,31]. Finally, due to its extreme nature, VIPA may not be suitable for everyone and, apart from a few experimental studies, might not be adopted for enough time to show effects on MDD and GAD in the general population.

One strength of this study was the exploration of the association between physical activity and individual symptoms of depression/anxiety, as mental health symptoms may differ in their etiology, risk factors, impairment, etc, and thus may show differential associations with physical activity [11,25]. Analyzing individual symptoms and their causal associations is an initial step toward personalized treatment of mental health disorders that recognizes the heterogeneity of MDD and GAD [11]. A previous study explored the association between individual symptoms of MDD and physical activity and found that eight out of 26 symptoms were relevant in young adults diagnosed with MMD [25].

This study was limited by its cross-sectional design, which prevented the analysis from generating the direction of the association between physical activity and the risks of MDD and GAD. The study was also limited by its bivariate analysis, which may be affected by other behavioral factors. However, it provided initial insights on the effect of physical activity on mental health in a general community setting in Saudi Arabia, which adds to the global literature in this area of research. The findings of this study may be relevant to other countries with high levels of physical inactivity like Saudi Arabia, but generalization of the results to other countries may be limited.

### Conclusion

This study found that fulfilling guidelines on MIPA (150 min/week) is associated with lower depression scores among male participants and generally fewer depressive and anxiety symptoms. Increasing the general population’s awareness of the need to increase moderate physical activity levels may improve the population’s mental health.

## Abbreviations

GAD: generalized anxiety disorder
GAD-7: Generalized Anxiety Disorder-7
MDD: major depressive disorder
MIPA: moderate-intensity aerobic physical activity
PHQ-9: Patient Health Questionnaire-9
VIPA: vigorous-intensity aerobic physical activity
WHO: World Health Organization
